# H2S Donor and Bone Metabolism

**DOI:** 10.3389/fphar.2021.661601

**Published:** 2021-07-22

**Authors:** Yanming Hao, Hongzhen Wang, Lingna Fang, Jinsong Bian, Yan Gao, Chong Li

**Affiliations:** ^1^ Department of Orthopedics, the First Peoples’ Hospital of Kunshan, Kunshan, China; ^2^ Department of Endocrinology, the First Peoples’ Hospital of Kunshan, Kunshan, China; ^3^ Department of Pharmacology, Southern University of Science and Technology, Shenzhen, China

**Keywords:** bone metabolism, mechanisms, diseases, hydrogen sulfide, donor

## Abstract

Hydrogen sulfide (H2S) has been recognized as the third gasotransmitter, following nitric oxide and carbon monoxide, and it exerts important biological effects in the body. Growing evidence has shown that H2S is involved in many physiological processes in the body. In recent years, much research has been carried out on the role of H2S in bone metabolism. Bone metabolic diseases have been linked to abnormal endogenous H2S functions and metabolism. It has been found that H2S plays an important role in the regulation of bone diseases such as osteoporosis and osteoarthritis. Regulation of H2S on bone metabolism has many interacting signaling pathways at the molecular level, which play an important role in bone formation and absorption. H2S releasing agents (donors) have achieved significant effects in the treatment of metabolic bone diseases such as osteoporosis and osteoarthritis. In addition, H2S donors and related drugs have been widely used as research tools in basic biomedical research and may be explored as potential therapeutic agents in the future. Donors are used to study the mechanism and function of H2S as they release H2S through different mechanisms. Although H2S releasers have biological activity, their function can be inconsistent. Additionally, donors have different H2S release capabilities, which could lead to different effects. Side effects may form with the formation of H2S; however, it is unclear whether these side effects affect the biological effects of H2S. Therefore, it is necessary to study H2S donors in detail. In this review, we summarize the current information about H2S donors related to bone metabolism diseases and discuss some mechanisms and biological applications.

## Introduction

With the development of aging society, the incidence of bone metabolic diseases is increasing year by year, such as osteoporosis and osteoarthritis, which seriously affects the quality of life of the elderly. Osteoporosis is a systemic bone disease characterized by osteopenia, destruction of bone microstructure and decrease of bone strength, resulting in the increase of bone fragility and fracture. Osteoblasts and osteoclasts play an important role in the process of bone metabolism. At present, the main mechanisms of osteoporosis drugs include inhibiting bone resorption, promoting bone formation, other mechanisms and traditional Chinese medicine, such as bisphosphonates, estrogen and parathyroid hormone (PTH), etc. However, long-term use of bisphosphonates can lead to jaw necrosis and atypical fractures of long bones (Hollick and Reid, 2011). Recombinant thyroid hormone (PTH) has been shown to directly promote bone formation, but at the same time stimulate osteoclast production and increase bone resorption (Wang et al., 2007). The large use of estrogen replacement therapy and its analogues will increase the risk of cardiovascular events or breast cancer in osteoporotic patients (Thorbjarnardottir et al., 2014). Osteoarthritis is a kind of joint degenerative disease with joint pain, deformity and dysfunction, which is the most common form of arthritis and the main cause of disability in the elderly. Although a variety of metabolic, genetic, epigenetic and local factors are involved, the complete etiology is still unclear. At present, there is no cure for this kind of pathology, and we are actively looking for alternative treatments to delay or prevent its progress. Therefore, it is of great clinical significance to find a new treatment.

As a newly discovered gaseous signaling molecule, H2S plays an important role in the cardiovascular, nervous, metabolic, neuroendocrine, and bone metabolism systems (Wang, 2010). In mammalian tissues, H2S is synthesized endogenously by three tissue-specific enzymes: cystathionine *γ*-lyase (CSE), cystathionine β-synthase (CBS), and 3-mercapto-pyruvate sulfurtransferase (3-MST) (Singh et al., 2009). The first two enzymes are pyridoxal 5-phosphate (PLP)-dependent, using L-cysteine (Cys) and homocysteine (Hcy) as substrates and are catalyzed by CBS and CSE; the third is in the pyridoxal-5′-phosphate (PLP)-independent 3-mercaptopyruvate sulfurtransferase (MST) pathway, which uses sulfur-containing amino acids as substrates (Gallego et al., 2008). The metabolic pathway of H2S in the body operates through the oxidation of mitochondria to produce sulfate and thiosulfate, the final products of which are ammonium and pyruvate (Olson, 2012). To generate enough Adenosine triphosphate (ATP) from the mitochondrial oxidation of H2S, sufficient exogenous H2S needs to be present. Endogenous hydrogen sulfide increases the production of ATP by promoting S-sulfhydration of the mitochondrial inner membrane protein ATP synthase, and supplementation of exogenous hydrogen sulfide donor NaHS can also promote the production of ATP (Módis et al., 2016). In animals, H2S has been proved to be the electron source of mitochondrial ATP synthesis. At lower concentrations, H2S can supply electrons in the electron transport chain, thus increasing the yield of ATP (Bouillaud et al., 2013; Helmy et al., 2014). H2S is the endogenous activator of ATP-dependent potassium channel (KATP channel) (Módis et al., 2013). Excess H2S in the body can be removed by molecules containing metals and sulfides, such as peroxides, catalase, and glutathione oxides (Wang, 2012). It is worth noting that catalytic oxidation of H2S is the main mechanism for the regulation of H2S levels in cells (Hildebrandt and Grieshaber, 2008).

The lipid-soluble capability of H2S is five times that of its watevr solubility; therefore, H2S can pass quickly through the cell membranes without the need for any specific transporter or receptor. H2S promotes a number of cellular signals that regulate neurological, cardiac, and respiratory functions, as well as cell metabolism and survival (Mathai et al., 2009). The level of hydrogen sulfide in mammals still remain controversial, various reports describe H2S in the blood of mammals, mainly in the range of micromolar concentrations. In healthy animals and humans, the physiological range of H2S in circulation is estimated to be 10–100 μmol (Módis et al., 2013). Recent research have shown that the physiological concentration of H2S in the peripheral blood ranges from 30 to 100 μmol/L, and the H2S level in the mammalian brain is 50–160 μmol/L (Sunzini et al., 2020). At present, there is no direct evidence on the level of endogenous hydrogen sulfide in bone tissue. Only Burguera et al. have reported the biosynthesis of H2S in articular cartilage. The concentration of hydrogen sulfide in cartilage of osteoarthritis patients was 0.056 (0.016–0.080) nmol/g, and in healthy cartilage was 0.457 (0.296, 0.593) nmol/g (Burguera et al., 2020). In addition, Liu et al. found that BMMSCs expressed CBS and CSE, and produced H2S at the level of 8–10 μmol in the culture supernatant of BMMSC (Liu et al., 2014).

As a reductant, physiological concentrations of H2S exert antioxidant, anti-inflammatory, and anti-apoptotic properties (Kimura et al., 2010). Endogenous H2S in the plasma is negatively associated with aging and oxidative stress levels (Hine et al., 2015). Osteoporosis makes bones fragile and leads to an increased risk of bone fractures. Reactive oxygen species (ROS) are important factors during osteoporosis development (Manolagas, 2010). Studies have found that high levels of lipid peroxidation and low antioxidant enzyme activities are present in postmenopausal osteoporotic women and ovariectomized rats (Muthusami et al., 2005). Osteoblasts and osteoclasts play an important role in bone homeostasis, and osteoblasts are responsible for bone formation and mineralization, and bone resorption, respectively. In osteoporosis patients, the dynamic balance between bone resorption and bone formation is disrupted. ROS accelerate osteoblast apoptosis, inhibit their differentiation, and impair bone formation (Charman, 1989). To understand the biological function of H2S, it is necessary to carry out continuous research related to H2S. To study the mechanism and function of H2S, the H2S donor is an important research tool, and the H2S releasing ability of each donor type is quite different, which may lead to different results. A large number of H2S donors have been studied because of their therapeutic potential. Here, we summarize several H2S donors and their effects on bone metabolism (Table 1).

**TABLE 1:** Summary of H2S donors.

H2S donor	H2S release mechanism	Effects on bone metabolism	References
Sulfide salt	Rapidly hydrolysis	Promote osteoblast proliferation	Xu et al. (2011)
		Protect osteoblasts from oxidative stress-induced cell damage	
		Stimulate the transcription level of osteocalcin	
GYY4137	Slowly hydrolysis, pH-dependent	Prevent DEX-induced bone loss, stimulate the formation and differentiation of osteoblasts	(Xu et al., 2011), (Grassi et al., 2016)
Garlic-derived sulfur compounds	Thiol activation	Protect osteoblasts from ROS formation	(Qiu et al., 2015), [Ahmadian et al. (2017)], (Mukherjee et al., 2006c)
		Increases the activity of alkaline phosphatase and Ca2+ ATPase	
Dithiolethiones and their NSAID hybrids	Hydrolysis	Anti-inflammation and inhibit bone defects and bone loss	Herrera et al. (2015)
DM-22	Thiol activation	Inhibits H-OCS function and promotes osteogenic differentiation of H-MSCs	Martelli et al. (2012)
Sulfide synthesis from traditional Chinese medicine donor	Slowly hydrolysis	Reduce the production of active oxygen	Yan et al. (2017)
HNO donors	Hydrolysis	Produce anti-inflammatory effect	Zhou et al. (2016)
		No biological effects on bone metabolism were reported to date	
Photolabile H2S donors	Light activation	No biological effects on bone metabolism were reported to date	Fukushima et al. (2014)
Self-immolate thiocarbamates	Esterase-triggered	No biological effects on bone metabolism were reported to date	Steiger et al. (2016)

## H2S Donors

### Sulfide Salt with Bone Metabolism

Inorganic sulfurized salts, such as sodium sulfide (Na_2_S) and sodium H2S (NaHS), are widely used as H2S equivalents in research, as well as being the earliest donors of H2S studied. By using these sulfurized salts, some biological functions regulated by H2S have been determined (Whiteman et al., 2010).

The most common H2S donors are sodium sulfide and sodium hydrosulfide (Jimenez, 2010), which can be used to prepare H2S standard solutions. Sodium sulfide has the advantage of rapidly increasing the concentration of H2S. In addition, they are “clean” donors, because they do not form by-products after the release of H2S. NaHS improves cell viability, reduces apoptosis induced by H_2_O_2_, and promotes osteoblast proliferation by enhancing the transcription and activity of alkaline phosphatase in MC3T3-E1 osteoblasts. In addition, NaHS treatment can also stimulate the transcription level of osteocalcin, the main bone matrix protein, and collagen protein expression, the main component of bone tissue. NaHS reverses the decrease in superoxide dismutase activity, active oxygen production, and nicotinamide adenine dinucleotide phosphate (NADPH) oxidase activity in osteoblasts treated with H_2_O_2_. H2S protects osteoblasts from oxidative stress-induced cell damage and proliferation and differentiation inhibition through mitogen-activated protein kinase (MAPK) (P38 and ERK1/2)-dependent mechanisms; therefore, H2S may have potential therapeutic value for osteoporosis (Xu et al., 2011). In bone loss induced by hyperhomocysteinemia (HHcy), NaSH epigenetically mitigates bone loss through osteoprotegerin (OPG)/receptor activator of nuclear factor kappa-B ligand (RANKL) regulation (Behera et al., 2018a). Studies report that NaHS decreased the differentiation of human osteoclasts in a dose-dependent manner by up-regulating the expression of nuclear factor erythroid 2-related factor 2 (NRF2) protein (Gambari et al., 2014). The activation of NRF2 may inhibit the differentiation of human osteoclasts by activating the continuous antioxidant response of osteoclast precursors (Gambari et al., 2014). In addition, NaHS can reduce cortical bone loss caused by spinal cord injury, probably by reducing oxidative stress, inhibiting matrix metalloproteinases (MMPs) activity and activating Wnt/β-catenin signal transduction (Yang et al., 2017).

NaHS intervention also has an effect on metabolic osteoporosis caused by CBS deficiency. Cysteine β-amylase heterozygote knockout mice were fed a methionine-rich diet, and the NaHS donor was supplemented for 8 weeks. NaHS treatment was used to normalize the plasma H2S and completely prevent trabecular bone loss in CBS+/-mice. The recovery of H2S may provide a treatment for metabolic osteoporosis caused by CBS deficiency (Behera et al., 2018b).

However, one of the main problems of such donors is that the release of H2S is uncontrollable. Once the sulfate dissolves in the aqueous solution, it releases H2S immediately and completely. A large number of rapidly released H2S causes a rapid drop in blood pressure and toxic effects in patients and animals (Li et al., 2008).

### GYY4137 and Phosphorodithioate-Based Donors with Bone Metabolism

The morpholin-4-ium 4 methoxyphenyl (morpholino) phosphinodithioate (GYY4137) is a novel water-soluble donor of H2S, which can release H2S slowly, sustaining a controlled release. Some experiments showed that 4.17 ± 0.5 nmol/25 min of H2S was released from 1 mm of GYY4137 (Zhao et al., 2015), and the mechanism of release was likely through hydrolysis. In addition, the release is pH-dependent; studies showed signs of maximum release at acidic pH (3.0) and less release at neutral or basic pH (7.4, 8.5) (Martelli et al., 2013). After administering GYY4137 to rats, the concentration of H2S in the plasma reached its highest level after 30 min and lasted for 3 h, indicating that GYY4137 released H2S much more slowly than NaHS. Cytotoxicity tests in rats showed that GYY4137 did not cause significant cell damage for three days at doses up to 100 μM, while NaHS caused apoptosis in smooth muscle cells at similar doses and times (Baskar et al., 2007). The difference in the experimental results may be explained by the different release rates of the two H2S donors. The main advantage of GYY4137 is that H2S can be slowly released.

GYY4137 plays a role in glucocorticoid-induced bone loss. By supplementing GYY4137 with H2S, bone formation can be promoted and bone absorption can be inhibited to prevent dexamethasone (DEX)-induced bone loss. It can also prevent osteoporosis caused by DEX and prevent trabecular and cortical bone loss. The mechanism of action can be achieved by activating the Wnt/β-catenin signaling pathway (Ma et al., 2019). GYY4137 activates Wnt signal by increasing the production of Wnt ligands Wnt16, Wnt2b, Wnt6 and Wnt10b in bone marrow, thus increasing the formation of osteoblasts (Grassi et al., 2016). In addition, GYY4137 can enhance osteoblast differentiation by stimulating the transcription level of runt-related transcription factor 2 (Yang et al., 2019a). GYY4137 also protected osteoblasts from apoptosis induced by DEX. These results indicate that GYY4137 is an effective way to prevent and treat osteoporosis and osteonecrosis caused by glucocorticoids (GCs) (Ma et al., 2019). In the fracture model, GYY4137 can antagonize the loss of calcium and phosphorus by reducing glucocorticoid secretion and inhibiting the vulcanization of glucocorticoid receptor α (Liao et al., 2021).

GYY4137 can also stimulate the formation and differentiation of osteoblasts through an ERK1/2-dependent antioxidant mechanism and successfully inhibit the oxidative damage of MC3T-E1 induced by hydrogen peroxide (Xu et al., 2011). It has been found that GYY4137 can prevent particle-induced inflammation and osteolysis through SIRT1 pathway during prosthesis loosening (Liu et al., 2020). GYY4137, as a controlled-release donor of H2S, can simulate the formation of endogenous H2S and has more advantages than sodium sulfide as an exogenous donor (Liu et al., 2013). By intraperitoneal injection of GYY4137 as a supplement of endogenous H2S, the physiological level of H2S in rat plasma can be improved and maintained to regulate the balance of calcium and phosphorus metabolism. The results showed that GYY4137 was a better intervention drug than alendronate and had a certain therapeutic effect on osteoporosis after oophorectomy (Xu et al., 2018).

### Natural Sulfur-Containing Compounds with Bone Metabolism

Garlic contains rich fat-soluble and water-soluble sulfides and can inherently release H2S, which has a protective effect against postmenopausal osteoporosis. H2S donors derived from garlic include diallyl sulfide (DAS), diallyl disulfide (DADS), and diallyl trisulfide (DATS) (Amagase, 2006). DAS and s-DADS are the main components of garlic with high antioxidant activity. DADS protects osteoblasts from ROS formation and protects smokers *via* ROS formation (Qiu et al., 2015). According to previous studies, these sulfur-containing compounds could inhibit the downregulation of the c-Jun N-terminal kinase (JNK) signaling pathway, which may lead to the protective role of S-allyl cysteine in oxidized-LDL formation and inhibition of nuclear factor-kappa B activation by hydrogen peroxide (Malandrino et al., 2012; Dada et al., 2014).

Garlic and its derivatives can be beneficial for osteoporosis to inhibit bone absorption, and it might also have a significant influence on carbonyl groups, advanced oxidation protein products (AOPPs), and ROS. It is likely that garlic prevents osteoporosis by suppressing oxidative stress. Previous research has shown that an increased oxidative stress index was observed in the garlic-treated group, but it was significantly reduced in the carbonyl group and AOPPs after garlic administration (Meisinger et al., 2014; Ahmadian et al., 2017).

The comparison of the anti-osteoporosis potential between garlic and 17-β-estradiol showed that garlic has the function of preventing and maintaining bone health. Garlic can reduce the excretion of calcium and phosphate and plays an active role in counteracting the increased osteoclast activity and bone absorption due to the lack of ovarian hormones. Garlic has potential antioxidant activity, which may be the main reason for scavenging free radicals produced by a trap. So far as potential is concerned, Garlic’s efficacy as a natural therapeutic agent must be valued. In fact, garlic may prove therapeutically better than estrogen when comparing the well-established negative side effects of the use of estrogen as an anti-osteoporotic agent (Mukherjee et al., 2006a). Garlic can significantly increase the calcium transfer of all intestinal segments of bilaterally ovariectomized rats and increases the activity of alkaline phosphatase and Ca^2+^ ATPase (Mukherjee et al., 2006b).

DATS and DADS, the donors of H2S, are the two main organic sulfides in garlic oil, which are allicin produced by garlic decomposition, and their health benefits have been widely studied. Garlic tablets not only have a good effect on postmenopausal osteoporosis but also have many beneficial effects on oxidative stress, and can reduce the activity of NADPH oxidase and the expression of vascular cell adhesion molecule 1 (VCAM-1), thereby reducing inflammation. DADS can attenuate inflammatory osteolysis by inhibiting osteoclast formation through NF- κ B-NFATc1 signal pathway (Yang et al., 2019b). In patients with arthritis, DADS inhibits the expression of three MMPs induced by IL-1/OncostatinM (OSM) in a dose-dependent manner, thus protecting articular cartilage (Williams et al., 2010).

Both chemical management and estrogen therapy have side effects. The advantage of allicin is its inhibitory effect on oxidative stress; other food extracts of onion, ginger, Nigella sativa, and Berberis vulgaris can also be further studied.

### 1,2-Dithiole-3-Thiones and H2S Hybrid NSAIDs with Bone Metabolism

1,2-disulfide-3-thioketone (DTT) is recognized as the core of H2S release; it releases H2S in aqueous solution, and hydrolysis may be the mechanism of H2S production by DTT (Hagle and McKay, 1996). Elemental sulfur or phosphorus pentasulfide is used for the dehydrogenation and sulfurization of allyl methyl to obtain the desired product. In another method, disulfide acids are obtained from the reaction between ketones and carbon disulfide (CS_2_), react with hexamethyl disulfide (HMDT, sulfur resource), and N-chlorosuccinimide (NCS, an oxidant) is replaced by DTTs. In addition, β-keto esters react with the Lawesson reagent to form DTTs (Kianmehr et al., 2010).

The mixed NSAIDs (HS NSAIDs) produced by DTT combined with NSAIDs significantly reduced gastrointestinal damage compared to maternal NSAIDs (Sparatore et al., 2011; Chan and Wallace, 2013; Gargallo and Lanas, 2013). HS NSAIDs are usually synthesized by coupling NSAIDs with 5-(4-hydroxyphenyl)-3h-1,2-disulfide-3-thioone (ADT-OH) in the presence of dicyclohexyl carbodiimide (DCC) and 4-dimethylaminopyridine (DMAP). When heated to 120°C in dimethyl sulfoxide (DMSO) phosphate buffer mixture, DTT releases H2S, which indicates that DTT can be regarded as a hydrolytic H2S donor (Qandil, 2012), and is often used to prepare mixed drugs of H2S donors. Some of the main candidate drugs have been reported in the fields of inflammation, the cardiovascular system, urinary system, and neurodegenerative diseases. These mixtures show significant improvement in activity and side effects, suggesting that H2S has useful pharmacological effects (Sparatore et al., 2011); rats receiving the same amount of acs14 did not show gastric injury, and the concentration of H2S in plasma increased three times.

ATB-346, a derivative product of H2S releasing naproxen, can significantly inhibit bone defects and bone loss caused by ligation. The H2S releasing part of the ATB-346 compound not only does not damage the effect of naproxen on periodontitis, but also improves bone quality, which provides a potential new adjuvant therapy for the treatment of periodontal diseases (Herrera et al., 2015).

Although HS-NSAIDs have been shown to play a role in H2S release in many tissues and organs, the mechanism of release of H2S *in vivo* needs to be further studied. Additionally, whether the biological effect of HS-NSAIDs is related to H2S or sulfas needs to be further studied.

### Mercaptan-Activated H2S Donor with Bone Metabolism

Mercaptan-activated H2S donors were first reported in 2011, and are unstable and easily broken to form a hydrosol anion, which is the main form of H2S under physiological conditions. The acyl group was used to protect the -SH residue of N-SH. The resulting compound, N-(benzoylthio) benzamide, was stable in water only in the presence of thiols (cysteine or glutathione). These donors were evaluated in plasma, and similar H2S release products were observed (Zhao et al., 2011).

DM-22, a new H2S hybrid n-bps synthesis, can induce the osteogenic differentiation of mesenchymal stromal cells human mesenchymal stem cells (H-MSCs) and maintain anti-osteoclast activity *in vitro* (Martelli et al., 2012). DM-22 is a slowly released H2S donor derived from a combination of alendronate (AL) and aryl isothiocyanates that release a portion of H2S. It is cultured in an aqueous solution (pH 7.4 and 20°C analysis buffer) for 1 mm, resulting in the production of a time-dependent increase in H2S concentration in the presence of 4 mm L-cysteine. H2S production rises to a stable state after 10 min and reaches a maximum H2S concentration of 42.0 ± 2.1 M after 20 min. Conversely, in the absence of l-cysteine, incubation of DM-22 (1 mm) resulted in a significant reduction in H2S levels, which is dependent on the H2S release mechanism of organic mercaptan (Rapposelli et al., 2017). The effects of DM-22 and AL on the osteogenic differentiation of human osteoclasts (H-OCS) and H-MSCS were detected at a concentration of 1–33 nm *in vitro*. Current measurements show that DM-22 releases H2S at a slow and mercaptan-dependent rate. DM-22 significantly inhibited H-OCS differentiation and function, maintaining residual H-OCS activity even at high doses of 33 μm. Furthermore, DM-22 inhibited the differentiation and function of H-OCSs without causing cytotoxicity. DM-22 showed no cytotoxicity over the whole concentration range. The release of H2S by DM-22 has persistent kinetics, which conforms to the characteristics of the isothiocyanate-type H2S-donor (Martelli et al., 2013; Citi et al., 2014). In the design of this hybrid drug, this pharmacological property could be expected to improve the overall pharmacological characteristics and attenuate the possible cytotoxicity of the BPS molecular portion.

DM-22 is a novel cytokine that inhibits H-OCS function and promotes osteogenic differentiation of H-MSCs without cytotoxicity. It is an ideal candidate for a new family of bone anabolic drugs, which would provide new therapeutic opportunities in the field of bone loss.

### Sulfide Synthesis from Traditional Chinese Medicine Donor with Bone Metabolism

SDSS is a representative H2S donor synthesized by traditional Chinese medicine; it is a donor synthesized by Danshensu (DSS) and H2S, both of which have a significant antioxidant effects in different systems. ROS accelerate the apoptosis of osteoblasts, inhibit their differentiation and damage bones (She et al., 2014; Li et al., 2015). Danshensu [β-(3,4-dihydroxyphenyl) lactate] is an important water-soluble compound extracted from *Salvia miltiorrhiza*. It is widely used in the treatment of various microcirculatory disorder-related diseases in China (Yu et al., 2012). DSS can reduce inflammation and inhibit ROS formation (Jiang et al., 2015). In addition to these effects, DSS has a beneficial effect on bone formation. For example, previous studies have shown that DSS not only promotes osteoblast differentiation and bone matrix formation but also regulates osteoblast differentiation (Naghibi et al., 2014).

SDSS is also a slow-release donor of H2S. In rats, H2S is released from the plasma approximately 6 min after intravenous administration. It reaches its maximum value 15 min after administration and slowly decreases with time. SDSS can release H2S in MC3T3-E1 osteoblasts and restore the ability of H_2_O_2_ to inhibit the differentiation of osteoblasts, which is due to the stimulation of bone sialoprotein, runt-related transcription factor-2, collagen expression, alkaline phosphatase activity, and bone nodule formation. The study showed that SDSS could reduce the production of active oxygen induced by H_2_O_2_, play a protective role, and reverse the decrease in superoxide dismutase activity, decrease in glutathione content, and increase in ROS production in H_2_O_2_ treated cells. In addition, SDSS significantly reduced the activation of p38, ERK1/2, and JNK MAPKs induced by H_2_O_2_, and SDSS also stimulates the phosphatidylinositol 3 kinase/AKT signaling pathway, blocking the pathway and reducing the cell-protective effect of SDSS. Importantly, SDSS had no significant toxic effect on the apoptosis of MC3T3-E1 cells induced by H_2_O_2_ (Yan et al., 2017).

## Other H2S Donors

Besides the above-mentioned H2S donors, some other donors have been studied in other systems and diseases. However, these studies have not referred to bone metabolism, which provides us with research space and direction for further research.

### HNO Donors

H2S and NO have many similar functions, and it is speculated that a new hybrid with both H2S and NO donors will be more effective than either alone. Therefore, some hybrid compounds (such as NOSH-1) that release NO-H2S were prepared and tested (Kashfi, 2014). Bian et al. studied the biological function of HNO in microglia with Danggui salt, a donor of HNO (Zhou et al., 2016). The study of HNO and bone metabolism of H2S donors has not yet been carried out.

### Photolabile H2S Donors

The H2S released from the light-controlled H2S donor was directly proportional to the light intensity and duration. It is difficult to control the release rate of H2S from these donors because the formation of H2S depends on the hydrolysis of Gemini mercaptan in water. Some researchers want to develop a suitable light movable protecting group on H2S, which will provide an H2S donor that can be directly controlled by light; in other words, two protecting groups release H2S after photolysis, which releases H2S without producing any highly active substance and makes H2S completely dependent on the release of light (Fukushima et al., 2014). However, the biological application or pharmacological properties of these light-induced donors have not been reported.

### Self-Immolate Thiocarbamates

Self-immobilized carbamates release amines with payloads and extrude carbon dioxide as a by-product. The formation of thiocarbamates by replacing carbonyl oxygen with sulfur atoms leads to the release of carbonyl sulfur (COS). In the biological environment, COS is rapidly hydrolyzed by carbonic anhydrase (CA) to H2S and CO_2_. Carbonic anhydrase is a ubiquitous enzyme found in plants and mammalian cells. When COS is added to a deoxygenated aqueous solution containing cafrombovine red cells, H2S is produced rapidly by using the H2S response electrode (Steiger et al., 2016). However, none of these studies have focused on its biological and pharmaceutical effects.

## Endogenous H2S

Endogenous H2S is formed from cysteine and homocysteine by CBS, CSE, and 3-mercaptopyruvate in mammalian cells. Although CBS is predominant in the hippocampus and cerebellum, endogenous H2S protects against H_2_O_2_-induced cytotoxicity in MC3T3-E1 osteoblastic cells (Burguera et al., 2020). The experiment demonstrated that bone marrow mesenchymal stem cells (BMMSCs) produce hydrogen sulfide to regulate their self-renewal and osteogenic differentiation, while deficiency of hydrogen sulfide results in defective differentiation of BMMSCs. H2S deficiency leads to an abnormal influx of intracellular Ca^2+^ because it reduces the sulfhydrylation of cysteine residues on multiple Ca^2+^ TRP channels. This reduced Ca^2+^ flux downregulates PKC/ERK-mediated Wnt/β-catenin signaling, which controls the osteogenic differentiation of BMMSCs. Mice deficient in H2S exhibit an osteoporotic phenotype that can be restored by releasing small molecules of H2S (Liu et al., 2014). These results suggest that H2S can regulate BMMSCs, which provides a possible treatment for osteoporosis and other diseases caused by H2S deficiency.

## Conclusion

H2S is a gaseous neurotransmitter that plays an important role in bone metabolism. Research on H2S and bone metabolism involves osteoblasts, osteoclasts, ovariectomized and hormone-induced osteoporosis models, bone metabolism models after spinal cord injury, H2S key enzyme gene knockout models, epigenetic animal studies, and even human clinical studies select different donors. The properties of donors should be carefully considered, including release rate, by-products, and cross-reactions with other biomolecules. Likewise, other technicalities must be considered, such as whether the release of H2S can be well controlled, the release mechanism of H2S, the by-products after H2S release, and the biological activity of these by-products as well as possible interference effects. Thus, we should be cautious and control the experiment carefully if using H2S donors, However, the development of H2S donors is crucial for understanding the biological function of H2S. We look forward to seeing more interesting research in this area.
